# Risk Factors for Progression in Vestibular Schwannomas After Incomplete Resection: A Single Center Retrospective Study

**DOI:** 10.3389/fneur.2021.778590

**Published:** 2021-11-26

**Authors:** Jiuhong Li, Xueyun Deng, Daibo Ke, Jian Cheng, Si Zhang, Xuhui Hui

**Affiliations:** ^1^Department of Neurosurgery, West China Hospital, Sichuan University, Chengdu, China; ^2^Department of Neurosurgery, Nanchong Central Hospital, The Second Clinical Medical College of North Sichuan Medical College, Nanchong, China

**Keywords:** vestibular schwannoma, incomplete resection, progression, risk factor, internal auditory canal, tumor size, residual tumor volume

## Abstract

**Background and Purpose:** The risk factors for progression in vestibular schwannomas (VSs) after incomplete resection (IR) remain to be elucidated. The purpose of this study was to investigate the risk factors for progression in remnant VSs after surgery.

**Methods:** From January 2009 to January 2018, 140 consecutive patients who underwent IR of VSs *via* suboccipital retrosigmoid approach in our institution were retrospectively analyzed. During follow-up, if progression was detected, the patient was classified into Progressive Group (PG); if the residual tumor was stable or shrank, the patient was classified into Stable Group (SG). Univariate analysis and multivariate analysis were used to evaluate the risk factors for progression after IR of VSs.

**Results:** After a mean follow-up of 80.4 months (range, 24–134 months), 35 (25.0%) patients (PG) had a progression, and no progression was detected in 105 (75.0%) patients (SG). The average tumor size was 36.5 ± 8.9 mm in PG and 31.0 ± 9.8 mm in SG, respectively. The residual tumor volume was 304.6 ± 443.3 mm^3^ in PG and 75.9 ± 60.0 mm^3^ in SG, respectively. Univariate analysis showed that preoperative tumor size, residual tumor volume, and irregular internal auditory canal (IAC) expansion were significantly different between the two groups, whereas gender, age, cystic component, or Ki-67 labeling index (LI) did not differ significantly between the two groups. Multivariate analysis showed residual tumor volume was the independent risk factor for progression.

**Conclusions:** VSs that underwent IR with larger preoperative size, greater residual tumor volume, or irregular IAC expansion may have a higher progression rate. Strict follow-up with shorter interval in these patients to detect early progression is necessary.

## Introduction

Vestibular schwannoma (VS) is a common benign neoplasm originating from the VIII cranial nerve sheath, accounting for around 8% of all intracranial tumors and 80% of cerebellopontine angle tumors (1–3). Surgical resection remains to be an optimal treatment regarding VSs, especially in large (≥ 3 cm) VSs (4). Incomplete resection (IR) may be encountered even for an experienced neurosurgeon due to many factors such as severe adhesion between tumor and peripheral neurovascular structures (5). Furthermore, to preserve favorable neurological function, especially facial nerve (FN) function, strategies including IR with adjuvant stereotactic radiosurgery (SRS) for regrowing tumor and planned partial resection (PR) followed by SRS have been proposed (6, 7). Overall, the IR rate of VSs ranges from 2.1 to 49.2% in the literature (8–12). Although VS is a kind of benign neoplasm with slow growth rate, it has the potential for recurrence/regrowth regardless of any extent of resection (EOR), especially in patients with VS receiving IR. According to the literature, VSs have a different recurrence/regrowth rate when they receive different EOR, with 2.4–3.4%, 3–29%, 23.8–52%, and 25–62.5% progression rate after total resection (TR), near total resection (NTR), subtotal resection (STR), and partial resection (PR), respectively (12–17). Thus, patients with VSs who underwent IR had a higher progression (regrowth) rate compared with those who received TR. However, only part of patients with VSs who underwent IR have progression (12).

The literatures on predicting the progression of VSs after surgical resection are limited, mostly focusing on clinical and histopathological parameters, such as age, sex, preoperative tumor size, tumor cystic formation, and Ki-67 labeling index (LI) (8, 12, 17). Risk factors predicting the regrowth of remnant VSs after IR remain to be elucidated.

Therefore, we retrospectively analyzed the patients with VSs who underwent IR in our hospital from January 2009 to January 2018. According to the follow-up results, they were divided into Progressive Group (PG) and Stable Group (SG). We originally investigated the prognostic role of internal auditory canal (IAC) in remnant progression of VSs. To the best of our knowledge, this is one of the largest retrospective cohort work of VSs that underwent IR, and there is no literature exploring the correlation between the type of IAC and tumor progression after surgery (18).

## Methods

Patients with pathological confirmation of VS who underwent IR *via* suboccipital retrosigmoid approach from January 2009 to January 2018 were retrospectively enrolled in our study. The protocol was approved by the ethics committee of our institution. Written informed consent was obtained from all the patients or their authorized trustees and it was in accordance with the ethical guidelines of the Declaration of Helsinki.

### Inclusion Criteria

(1) Patients were pathologically confirmed with VS.
(2) The medical record of the EOR was IR.
(3) Post-operative brain-enhanced magnetic resonance imaging (MRI) performing 3 months after surgery confirmed the presence of residual tumor.
(4) Patient had at least one subsequent brain-enhanced MRI after initial radiological confirmation of tumor remanence.

### Exclusion Criteria

(1) Patients diagnosed as having neurofibromatosis II.
(2) Patients had a history of surgery for VS.
(3) Patients received radiotherapy of remnant VS before tumor progression was detected.
(4) No brain-enhanced MRI was conducted within 6 months postoperatively.
(5) Follow-up time was <2 years.

### Grouping Criteria

The tumor progression was defined as tumor enlargement of ≥2 mm compared with the residual tumor. During follow-up, if progression was detected, the patient was classified into the Progressive Group (PG); if the residual tumor was stable or shrank, it was classified into Stable Group (SG).

### Data Collection

Data including age, gender, size, cystic formation, IAC type, residual tumor volume, and Ki-67 LI performed on first operation were retrospectively collected.

**Figure 1:**
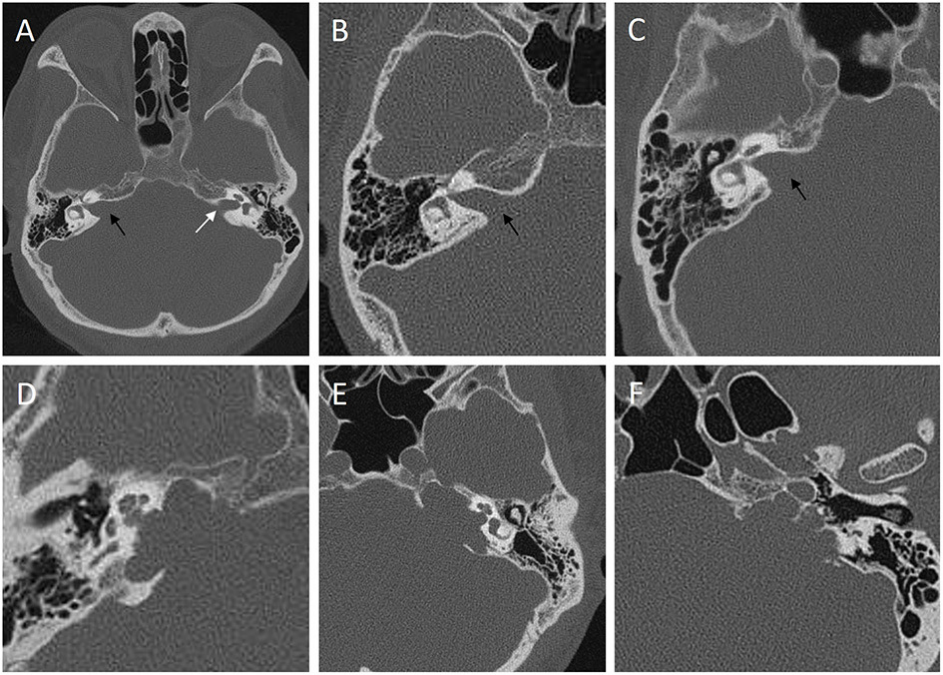
Classification of internal auditory canal (IAC) into regular type **(A–C)** and irregular type **(D–F)** according to the preoperative thin-slice CT bone window image of the temporal bone of the lesion side. **(A)** the right IAC is horn-shaped damaged type (black arrow) caused by a VS compared with the normal side (white arrow). **(B)** no expansion of the IAC (black arrow). **(C)** horn-shaped damaged type (black arrow). Curved damaged groove **(D,E)** or angled damaged groove **(F)** of the IAC wall could be observed in irregular IAC type.

(1) The tumor size was calculated as the maximal diameter on preoperative axial, sagittal, or coronal MRI performed before first operation.
(2) Cystic formation: we termed cystic VSs as where those diameters of cystic portion on enhanced MRI were larger than 50% of the whole tumor and those VSs without cystic formation or diameter of cystic portion ≤ 50% of the whole tumor were termed as solid VSs.
(3) Internal auditory canal type: we divided the IAC type into the following two types according to the preoperative thin-slice CT bone window image of the temporal bone of the lesion side: horn-shaped damaged or no expansion of IAC were termed as regular IAC type (Figures 1A–C); curved or angled damage of the IAC wall was termed as irregular IAC type (Figures 1D–F).
(4) Residual tumor volume: we calculated residual tumor volume by segmentation (multiplying the area of each part by the interval thickness and by adding all the interval volumes of each part.) according to enhanced MRI within 3–6 months after surgery.

### Follow-Up and Therapeutic Strategy

Follow-up was performed at 3 and 6 months, and then once a year after surgery. During the follow-up period, tumor progression was monitored by enhanced MRI. For patients who underwent IR, we did not perform immediate treatment. Only when progression was detected, we should perform one of the following treatments based on the tumor size, location, and the willingness of the patient: (1) close wait-and-see strategy, (2) gamma-knife radiosurgery, and (3) second operation. The indication for prescribing radiotherapy of remnant VSs was tumor enlargement on radiological examination while the tumor is relatively small ( ≤ 3 cm); the indication for reoperation of regrowing VSs was progressive tumor enlargement radiologically, with or without new symptoms related to the tumor. The whole screening process was performed by two authors (JHL and XYD) independently according to the inclusion and exclusion criteria. In case of any disagreement, a third author (DBK) was consulted.

### Statistical Analysis

All statistical analysis was performed with SPSS version 23 software (SPSS, Chicago, IL, USA). The Student's *t*-test was used to compare continuous variables. Pearson's chi-square test or Fisher's exact probability test was used to compare categorical variables. Univariate and multivariate logistic regression analyses were used to analyze gender, age, preoperative tumor size, cystic formation, ICA type, residual tumor volume, and Ki-67 LI between the two groups so as to seek the risk factors for the progression of residual VSs after IR. *p* < 0.05 were considered statistically significant. The cutoff point was determined using the receiver operating characteristic (ROC) curve.

## Results

From January 2009 to January 2018, a total of 836 patients with sporadic VS received surgical resection in our institution by two independent neurosurgeons, among which 140 (16.7%) cases received IR *via* suboccipital retrosigmoid approach and met all the defined inclusion and exclusion criteria and were enrolled in our work. During an average follow-up time of 80.4 months (range, 24–134 months), a total of 35 cases (25.0%) had tumor progression (PG) and 105 cases (75.0%) had no sign of progression (SG). In PG, the mean regrowth interval was 54.2 ± 34.5 months (range, 10–120 months). In PG, 15 cases (42.9%) underwent reoperation, among which 10 cases were totally removed after reoperation, three cases still had remnant tumor and two cases had third operation; six patients (17.1%) had gamma-knife radiosurgery and reoperation, among which five cases were totally resected after reoperation, and one case still had remnant tumor; another 14 cases (40%) had gamma-knife radiosurgery alone, among which eight cases were stable, and six were shrank.

Of the 140 cases, 85 (60.7%) were women and 55 (39.3%) were men. There were 25 (71.4%) women and 10 (28.6%) men in PG and 60 (57.1%) women and 45 (42.9%) men in SG. The average age was 42.6 ± 13.0 years (range, 20–65 years) and 47.0 ± 14.5 years (range, 16–74 years) in PG and SG, respectively. Enhanced MRI data were obtained in 27 cases in the PG before first operation, with an average preoperative tumor size of 36.5 ± 8.9 mm (range, 22–58 mm); enhanced MRI data were obtained in 82 cases in the SG before operation, with an average tumor size of 31.0 ± 9.8 mm (range, 11–53 mm). Ten cases (37.0%) were cystic and 17 cases (63.0%) were solid in PG, and 29 cases (35.4%) were cystic and 53 cases (64.6%) were solid in the SG. Preoperative thin-slice CT of the IAC was obtained in 17 cases in PG, of which 10 (58.8%) cases were regular type and seven cases (41.2%) were irregular type; preoperative thin-slice CT of the IAC was obtained in 46 cases in the SG, of which 44 cases (95.7%) were regular type and only two cases (4.3%) were irregular type. The residual tumor volume was 304.6 ± 443.3 mm^3^ (range, 30–2,160 mm^3^) in PG and 75.9 ± 60.0 mm^3^ (range, 10–340 mm^3^) in SG. The Ki-67 LI was tested in 13 patients in PG, with an average value of 3.2 ± 2.8% (range, 1.0–10.0%); the Ki-67 LI was tested in 32 patients in SG, with an average value of 2.5 ± 2.0% (range, 0.5–8.0%). Ki-67 LI was tested in 10 patients receiving reoperation, with an average value of 5.0 ± 2.9% (range, 2.0–11.0%).

Independent sample *t*-test showed that preoperative tumor size was statistically larger in PG compared with SG (*P* = 0.014) and residual tumor volume was statistically greater in PG compared with SG (*P* < 0.001). Fisher's exact probability test showed the incidence of IAC irregular type in PG was significantly higher compared with SG (*P* < 0.001). Univariate analysis showed that there was no statistical difference between the two groups regarding age, gender, cystic formation, and Ki-67 LI. The detailed statistical results are shown in Supplementary Table 1.

**Figure 2:**
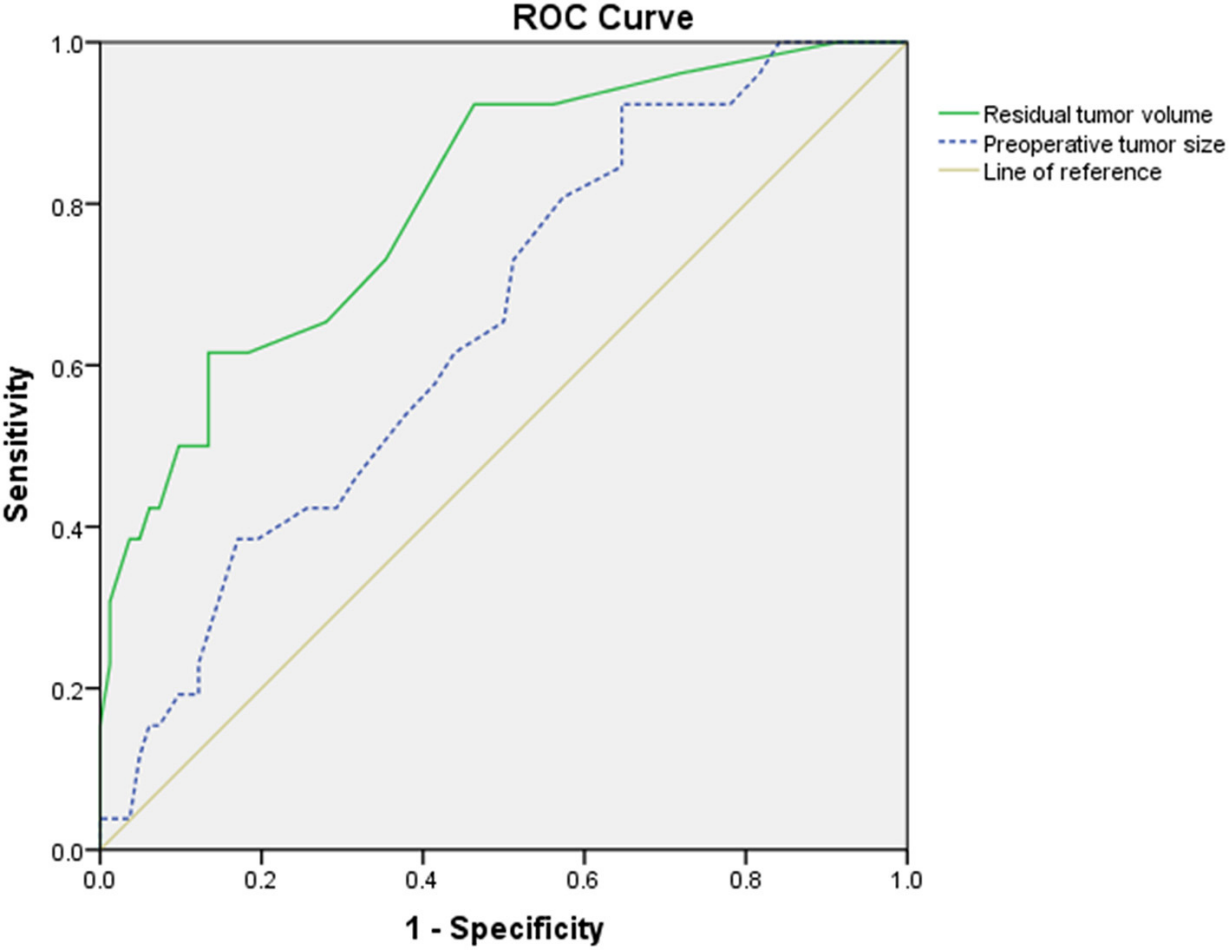
The receiver operating characteristic (ROC) curve of preoperative tumor size (blue dotted line) and residual tumor volume (green solid line). The area under the curve (AUC) was 0.651 and 0.805, respectively. The cutoff point of preoperative tumor size was 26.5 mm, with 0.923 sensitivity and 0.354 specificity, whereas the cutoff point of residual tumor volume was 95 mm^3^, with 0.615 sensitivity and 0.866 specificity.

The ROC curve showed that the optimal cutoff point of the preoperative tumor size was 26.5 mm, with 0.923 sensitivity and 0.354 specificity (Figure 2), and the cutoff point of residual tumor volume was 95 mm^3^, with 0.615 sensitivity and 0.866 specificity (Figure 2). The area under the curve (AUC) was 0.651 and 0.805, respectively (Figure 2). Multivariate regression analysis showed that residual tumor volume is an independent risk factor contributing the progression of remnant VSs (*P* = 0.012, OR = 1.017) (Supplementary Table 2).

## Discussion

The total removal rate of VSs is growing in many neurosurgery departments around the world. However, neurosurgeons sometimes have to compromise to IR for the sake of FN function preservation or because of the obstruction of bony structures, such as IAC wall. In surgical treatment of giant VS, we advocate that every effort should be made to achieve maximal resection. The essence of our institutional experience was that only if the neurophysiologic monitoring indicated or surgeons predicted a risk of facial nerve integrity and vulnerability, FN preservation trumped TR as a surgical goal (14). The fate of VSs that underwent IR includes regrowing, stable, or shrank (12, 19). According to the literature, VSs that received IR had approximately 10 times higher progression rate compared with those received TR (12–17). Besides, only part of remnant VSs would progress during follow-up. Therefore, it would be beneficial if we could find out the risk factors for regrowth in VSs receiving IR. Our study found that preoperative tumor size, residual tumor volume, and IAC type were significantly associated with regrowth in remnant VSs, although other clinical/radiological features, such as age, gender, cystic formation, and Ki-67 LI, did not have significant difference between the PG and SG. Multivariate analysis showed residual tumor volume was the only independent risk factor for progression.

### Clinical Risk Factors

Some investigators found that the growth rate of VSs in elderly patients is slower than that in younger patients (20–22). Our study did not find the predictive role of age and sex in remnant VSs, which is similar to the literature (8, 17, 23, 24). Overall, there is no strong evidence to prove that age and gender could be used as risk factors to predict regrowth of remnant VSs.

### Preoperative Radiological Parameters

Our results revealed that patients in the PG had significantly larger preoperative tumor size than those in the SG (*p* = 0.014), with AUC value of 0.651 (Figure 2), which means a low prognostic value. Our finding was compatible with the work of Bloch et al., who found that patients with greater remnant tumor had significantly higher rate of tumor progression and had significantly larger preoperative tumor size (12). The reasons may contribute to intrinsic faster growth rate in larger VSs (25, 26), or the larger VSs may have greater residual tumor, resulting in progression (24), although other researchers found that preoperative tumor size was not a significant contributory factor (8, 17, 23, 24). The discrepancy may contribute to the giant VSs (≥ 4 cm) which is more common in developing countries and different from developed countries (14), and larger mean tumor size may have a lower rate of TR. According to univariate analysis and ROC curve, we found that for remnant VSs, the preoperative tumor size >26.5 mm is a risk factor for higher rate of progression. Meanwhile, our multivariate analysis indicated that the residual tumor volume is the only significant risk factor for progression.

**Figure 3:**
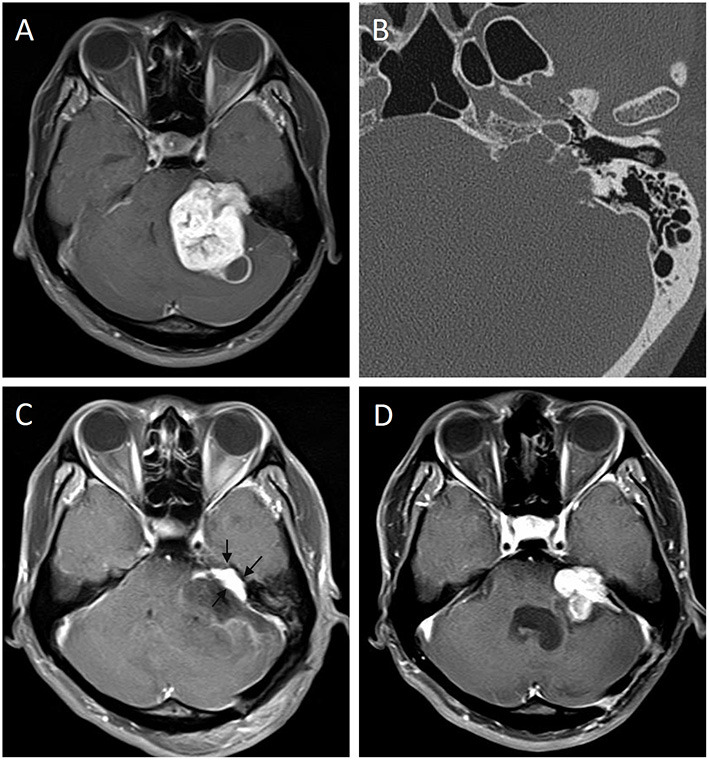
Progression of a remnant vestibular schwannoma with irregular IAC. Preoperative enhanced MRI **(A)** of a 29-year-old man showed a giant VS of left side. Thin-slice CT bone window image of temporal bone **(B)** showed irregular IAC type. Three-month postoperative enhanced MRI **(C)** showed remnant tumor located in the IAC (arrows). The tumor had progression 3 months later **(D)**.

Our study explored the correlation between the type of IAC and the progression of residual VSs for the first time. Univariate analysis showed that irregular destruction of the IAC is a risk factor for progression. We considered that the reason may be that it is more difficult to remove the tumor within the IAC because the tumor infiltrates in irregular corners of the IAC, which is hard to achieve adequate exposure of tumor, and thus gross TR is challenging (Figure 3). Also, the FN and bony structure of IAC may obstruct exposing and resecting tumors located in the bottom of IAC, resulting in greater amount of residuals, which leads to progression. However, multivariate analysis showed that IAC was not an independent risk factor for progression. We considered that if we further increase the sample size, the IAC type may be an independent risk factor for progression in remnant VSs. In summary, we recommended that patients with preoperative irregular damaged IAC should be informed that they may have a higher risk of progression if TR could not be achieved. The mechanism is unclear and requires more and larger samples of prospective, multicenter studies.

Some investigators pointed out that cystic VSs have higher proliferative activity than solid VSs (27), whereas our study found that there was no significant correlation between cystic formation and the rate of residual tumor progression. Chen et al. (18) found that for those VSs that underwent STR, six of 20 (30%) solid VSs had regrowth whereas only one of 18 (5.56%) cystic VSs had regrowth, but it did not reach statistical significance. Similarly, Fukuda et al. (17) did not find statistically significant correlation either. This was also consistent with the results of Hwang et al., who compared 15 cases of VSs with extremely rapid progression (>15 mm/year) with another 15 cases of non-progressive VSs and found no significant difference regarding cystic formation in the two groups (28). However, the sample size of the above studies is relatively small; thus, a prospective, multicenter study with a larger sample is necessary to explore whether cystic VSs have a significant impact on the progression of residual VSs compared with their solid counterparts.

### Post-operative Radiological Risk Factors

The best method to evaluate the amount of residual tumor is still controversial. Some scholars believed that there is no significant difference using residual tumor thickness or residual tumor volume (29), whereas others suggested using the latter because measurement errors may occur because of partial volume effect (30). Assessing the thickness of residual tumors is more intuitional and quicker; however, it is more dependent on interobserver variability. Thus, we used the residual tumor volume to evaluate the amount of residual tumors, which is more objective and easily calculated on tumor segmentation.

The mechanism of progression in remnant VSs remains to be clarified. The progression of the residual tumor may be determined by the characteristics of the tumor cells and the blood supply of the tumor. Breshears et al. retrospectively analyzed 66 patients who underwent STR, and greater residual tumor volume and residual tumor located at IAC predicted progression (8). Kasantikul et al. (31) believed that if the diameter of VS is >20 mm, the angiogenesis around the tumor would be significantly accelerated, and so the smaller residual tumor would benefit better tumor control. Fukuda et al. (17) found that the thickness of the residual tumor >7.4 mm was a risk factor for progression and residual tumor thickness was the only independent risk factor. According to univariate analysis and ROC curve, we found that residual tumor volume >95 mm^3^ was a risk factor for higher rate of progression, with a moderate predictive value of AUC 0.805 (Figure 2). Nevertheless, we hold that every effort should be made to achieve TR, which could achieve lower progression rate compared with IR.

### Histopathological Risk Factors

Ki-67 (MIB-1) LI, as a cell proliferation indicator, could be used to predict the progression of some intracranial benign tumors, such as meningiomas or pituitary tumors (32). Panigrahi et al. retrospectively analyzed 144 consecutive patients with sporadic VS, showing that the average Ki-67 LI was significantly higher in patients with recurrence at follow-up, and concluded that Ki-67 LI ≥ 3.5% at initial presentation was associated with recurrence regardless of the EOR (33). Another study found that tumor doubling time of VSs decreased logarithmically with increasing level of Ki-67 LI (34). Fukuda et al. (17) concluded that Ki-67 LI > 1.6% in patients with residual VSs is a risk factor for tumor progression. Hwang et al. (28) compared 15 cases of VSs with rapid progression (> 15 mm/year) after operation with 15 cases of stable VSs and found that Ki-67 LI in the rapid progression group was significantly higher than that in the stable group. According to our unpublished institutional data, the Ki-67 LI of patients with recurrent/regrowing VSs tested at reoperation was significantly higher than that of patients with stable VSs tested at the first surgery. Therefore, we hold that when VS progresses, Ki-67 LI, which reflects tumor proliferation characteristics, may subsequently rise. However, our findings showed that Ki-67 LI could not serve as a predictive factor of progression in remnant VSs. Overall, to determine whether Ki-67 LI could serve as a risk factor to predict progression of residual VSs, further study is warranted.

### Study Limitations

Our study has limitations. Firstly, this study is a retrospective non-randomized study with its intrinsic data selection bias. Secondly, this study is a single-center analysis of regrowth of VSs cases, which may be limited by the small sample size and may make the conclusion less convincing. Thirdly, our average follow-up time is 80.4 months (range, 24–134 months), which is insufficient to investigate whether VS has progressed. Fourthly, preoperative maximal diameter data could not be obtained in eight cases in the PG and 23 cases in the SG because the patients performed preoperative enhanced MRI in another hospital and the data could not be found, which could bring bias. Finally, Ki-67 LI was available in limited cases because it was not routinely performed before 2013 in our institution. Prospective, multicenter, and large sample size study together with clinicopathological and genetic analysis of VSs with irregular IAC is required.

## Conclusions

For patients with IR of VSs, a larger preoperative tumor size, a greater residual tumor volume, and irregular IAC type are the risk factors for progression. These patients require imaging scan with a shorter interval to early detect signs of tumor progression during follow-up.

## Data Availability Statement

The raw data supporting the conclusions of this article will be made available by the authors, without undue reservation.

## Ethics Statement

The studies involving human participants were reviewed and approved by Ethics Committee of West China Hospital of Sichuan University. Written informed consent to participate in this study was provided by all the patients or their authorized trustees. Written informed consent was obtained from the individual(s) for the publication of any potentially identifiable images or data included in this article.

## Author Contributions

SZ and XH: conception and design and study supervision. JL, XD, DK, and JC: development of methodology, writing, reviewing, and revisioning of the manuscript. JL, XD, DK, JC, and SZ: acquisition and analysis of data. DK and SZ: technical and material support. All authors have read and approved the manuscript. All authors contributed to the article and approved the submitted version.

## Funding

This work was supported by Youth Program of National Natural Science Foundation of China (No. 81801178), Program of Science & Technology Department of Sichuan Province (No. 2020YFS0222), and Program of Health Commission of Sichuan Province (No. 20PJ051).

## Conflict of Interest

The authors declare that the research was conducted in the absence of any commercial or financial relationships that could be construed as a potential conflict of interest.

## Publisher's Note

All claims expressed in this article are solely those of the authors and do not necessarily represent those of their affiliated organizations, or those of the publisher, the editors and the reviewers. Any product that may be evaluated in this article, or claim that may be made by its manufacturer, is not guaranteed or endorsed by the publisher.
