# Supplementary material for: Risk Factors for Progression in Vestibular Schwannomas After Incomplete Resection: A Single Center Retrospective Study
Source: Front Neurol. 2021 Nov 26;12:778590. doi: 10.3389/fneur.2021.778590 (PMC8660677; doi:10.3389/fneur.2021.778590)
Supplement: Supplementary file 1 [file Table_1.docx]

Table 1. Univariate logistic regression analysis for progression in VSs receiving incomplete resection

| Variables | Progression Group | Stable Group | Univariate analysis | |
| --- | --- | --- | --- | --- |
|  |  |  | *P* | OR (95% CI) |
| Gender, n (%) |  |  |  |  |
| Male | 10 (18.2) | 45 (81.8) | 0.134 | 0.533 (0.233-1.222) |
| Female | 25 (29.4) | 60 (70.6) |  |  |
| Age, yrs (mean ± SD) | 42.6 ± 13.0 | 47.0 ± 14.5 | 0.107 |  |
| Largest diameter, mm | 36.5 ± 8.9 | 31.0 ± 9.8 | 0.014 |  |
| Cystic formation, n (%) |  |  |  |  |
| Cystic | 10 (25.6) | 29 (74.4) | 0.875 | 1.075 (0.436-2.652) |
| Solid | 17 (24.3) | 53 (75.7) |  |  |
| Ki-67 LI, % | 3.2 ± 2.8 | 2.5 ± 2.0 | 0.328 |  |
| Internal auditory canal type, n (%) |  |  |  |  |
| Irregular damaged type | 7 (77.8) | 2 (22.2) | <0.001 | 14.700 (2.642-81.776) |
| Regular damaged type | 10 (18.5) | 44 (81.5) |  |  |
| Residual tumor volume, mm^3^ | 304.6 ± 443.3 | 75.9 ± 60.0 | <0.001 |  |
