# Supplementary material for: Risk Factors for Progression in Vestibular Schwannomas After Incomplete Resection: A Single Center Retrospective Study
Source: Front Neurol. 2021 Nov 26;12:778590. doi: 10.3389/fneur.2021.778590 (PMC8660677; doi:10.3389/fneur.2021.778590)
Supplement: Supplementary file 2 [file Table_2.DOCX]

Table 2. Multivariate logistic regression analysis for progression in VSs receiving incomplete resection

| Variables | Multivariate logistic regression analysis | |
| --- | --- | --- |
|  | *P* | OR (95% CI) |
| Largest diameter, mm | 0.393 | 4.981 (0.565-43.907) |
| Internal auditory canal type, n (%) |  |  |
| Irregular damaged type | 0.148 | 1.040 (0.951-1.137) |
| Regular damaged type |  |  |
| Residual tumor volume, mm^3^ | 0.012 | 1.017 (1.004-1.030) |
